# Fibroblastic reticular cells orchestrate long-term graft survival following recipient treatment with CD40 ligand–targeted costimulatory blockade

**DOI:** 10.1172/JCI165174

**Published:** 2022-12-15

**Authors:** Robert L. Fairchild

**Affiliations:** Department of Inflammation & Immunity, Lerner Research Institute, Cleveland Clinic, Cleveland, Ohio, USA.

## Abstract

Fibroblastic reticular cells (FRCs) maintain the architecture of secondary lymphoid organs, which optimize interactions between antigen-presenting dendritic cells and reactive naive T cells. In this issue of the *JCI*, Zhao, Jung, and colleagues investigated CD4^+^FoxP3^+^ regulatory T cell development and long-term heart allograft survival in recipients treated with peritransplant costimulatory blockade to inhibit CD40/CD40 ligand (CD40L) signaling. Treatment with an anti-CD40L monoclonal antibody (mAb) increased the lymph node (LN) population of Madcam1^+^ FRCs and altered their transcription profile to express immunoregulatory mediators. Administration of nanoparticles, containing the anti-CD40L mAb and a targeting antibody against high endothelial venules, delivered the treatment into LNs of allograft recipients. Direct LN delivery of the costimulatory blockade allowed decreased dosing and increased the efficacy in extending graft survival. The results provide insights into mechanisms by which FRCs can promote donor-reactive tolerance, and establish a strategy for administering costimulation-blocking reagents that circumvent systemic effects and improve allograft outcomes.

## Architecture of the immune system

The immune system is organized to maximize innate and adaptive immune responses to pathogen infection. Key features of this organization include lymphatic draining of cells and molecules from peripheral tissues into secondary lymphoid organs. In the lymph nodes (LNs), dendritic cells (DCs) that have acquired antigen and free antigen from infection sites are directed through afferent lymphatics into the T cell–rich paracortical regions. Importantly, naive T cells are directed from the vasculature to enter LNs through high endothelial venules (HEVs) and into the paracortical region where they scan immigrating antigen-presenting DCs. Those naive T cells that express a receptor reactive with the peptide/MHC complex on DCs, and engage costimulatory receptor ligands on the DCs, including CD80/86 and CD40, become activated to undergo clonal proliferation to generate the numbers of antigen-reactive T cells needed to eliminate the pathogen. Thus, the initiation of T cell–mediated immune responses requires intricate coordination: antigen-presenting DCs enter the LNs through afferent lymphatics, naive T cells maneuver through HEVs, and the two cell populations interact within the paracortical region.

The structural organization of the LN that guides the entry and trafficking of specific immune cells into distinct compartments depends on the function of a heterogeneous population of mesenchyme-derived cells, termed fibroblastic reticular cells (FRCs) (1, 2). Distinct populations of FRCs are distributed throughout the different LN compartments; as many as four different FRC populations have been identified in the T cell–rich paracortical region, suggesting there are different FRC functions within a given LN compartment (3, 4). The FRCs produce extracellular matrix fibers that support the LN architecture and position various immune cellular components into the different LN regions where they interact during the development of an immune response (5, 6). FRCs also produce chemokines, primarily CCL19 and CCL21, that direct naive T cell trafficking through HEVs and the DCs into the paracortical compartment (7, 8). The FRCs perform a third critical function in immune responses by interacting with resident DCs and those DCs entering the LN from the afferent lymphatics (9, 10).

## Peripheral tolerance

The induction and maintenance of peripheral tolerance to self-antigens is largely mediated by populations of CD4^+^FoxP3^+^ T cells and other regulatory T cells that inhibit activation of reactive T cells. Importantly, many strategies have been developed in preclinical models that induce and maintain peripheral tolerance to the introduction of exogenous antigens, including model protein antigens and allogeneic MHC molecules expressed on transplanted cells and organs. One of the most effective strategies involves the use of costimulatory blockade agents that do not interfere with T cell receptor engagement of peptide/MHC but inhibit delivery of costimulatory ligand signals required as second signals for reactive naive T cell activation (11, 12). One of these agents, CTLA-4Ig, blocks delivery of CD28-mediated costimulatory signals (13). Preclinical studies indicate that blockade of CD40-mediated costimulation, using CD40 ligand–blocking (CD40L-blocking) or CD40-blocking antibodies, may effectively achieve long-term allograft tolerance (14, 15). Moreover, administration of CD40-CD40L costimulation blockade reagents also promotes skewing of reactive T cell development to CD4^+^FoxP3^+^ T regulatory cells, which are required to maintain peripheral tolerance. Previous studies by the Bromberg and Adbi laboratories indicated that anti-CD40L monoclonal antibody–induced (mAb-induced) tolerance and the induction of T regulatory cells in allograft recipients depend on specific constituents of the recipient LN, suggesting this site as critical for initiation of alloantigen-specific tolerance (16–19). As with the activation of effector T cells, it is likely that the FRC-dependent structural properties of the LN are required for naive CD4^+^ T cell interaction with antigen-presenting DCs and their differentiation to regulatory CD4^+^FoxP3^+^ T cells.

## Interactions within the LN

In this issue of the *JCl*, Zhao, Jung, and colleagues have continued their extensive work investigating the role of LN constituents required for anti-CD40L mAb–induced tolerance to heart allografts in a mouse model (20). The authors used allograft recipients consisting of transgenic mice without LNs and mice expressing the diphtheria toxin receptor under the control of the CCL19 promoter to specifically deplete FRCs by treatment with diphtheria toxin. LNs were required for the CD40-CD40L–targeted tolerance, and FRC depletion at approximately 25 days after transplant collapsed the regulation that supported long-term allograft survival. These findings indicate that maintaining tolerance was dependent on the function of FRCs. Consistent with T cell skewing, in which T cells developed into regulatory cells rather than effector phenotypes in the LN following anti-CD40L mAb conditioning, the DCs within LNs of treated heart-allograft recipients expressed lower levels of class II MHC and costimulatory molecules CD80 and CD86 than LN DCs from nontreated recipients. Administration of anti-CD40L mAb in the absence of FRCs was accompanied by decreases in CD4^+^FoxP3^+^ T regulatory cells and DC trafficking into the LN, suggesting that FRC-DC interactions within the LN were critical for the immunoregulation induced by peritransplant anti-CD40L mAb treatment of heart-allograft recipients. These results further suggest an essential role for FRCs in maintaining the LN structure to organize DC interactions with naive T cells during the induction of tolerance, but do not preclude other potential FRC functions in the induction and/or maintenance of tolerance. The possibility remains that despite the absence of CD40, FRCs play a direct role in regulatory T cell development in the LN. Such a direct role was suggested by in vitro studies where cultured FRC lines promoted anti-CD3/anti-CD28 mAb–stimulated differentiation of CD4^+^ T cells to a CD4^+^FoxP3^+^ T regulatory phenotype, rather than to an effector CD4^+^ T cell phenotype. This skewing was consistent with studies indicating that FRC expression of self-peptide/class I MHC complexes induced tolerance of self-reactive T cells (20, 21).

Since FRCs constitute a heterogeneous population of LN-resident stromal cells, the peritransplant anti-CD40L mAb conditioning to induce tolerance would likely generate changes in specific FRC populations during the development and maintenance of tolerance. To test this hypothesis, the authors performed single-cell RNA sequencing of LN cells from allograft recipients treated with and without the peritransplant costimulatory blockade. They focused on five different LN stromal cell populations and observed a marked increase in Madcam1^+^ FRCs that expressed immunosuppressive mediators, including secreted frizzled-related protein 2, from the anti-CD40L mAb–treated recipients. The Madcam1^+^ FRCs also expressed higher levels of CCL19 and CCL21 that direct naive T cell entry into the LN. Overall, these RNA sequencing studies potentially identify an FRC mediator associated with the anti-CD40L mAb–induced prolonged allograft survival that might be useful as a biomarker and as a target to develop therapies to improve allograft function and survival in recipients treated with, at least, this costimulatory blockade reagent (20). Since FRCs serve as DC docking sites in the paracortical compartment, the change in Madcam1^+^ FRCs in anti-CD40L mAb–treated recipients raises the question as to the role of the DC in altering the transcripts and function of FRCs during tolerance induction. Mechanistically, critical crosstalk signaling likely evolves between FRC and DC interactions in the LN during the development of effector responses, and conversely, tolerance. Identification of these different signals may provide targets to boost or dampen immune responses as warranted.

Since the LN is a likely target of the anti-CD40L mAb–directed therapy, devising strategies to directly target the antibody to the LN might increase its efficacy and likely allow decreased dosing to avoid off-target effects of systemically administered antibody. Zhao, Jung, and authors confront the problem of systemic administration of anti-CD40L mAb–directed therapy by developing nanoparticles containing the anti-CD40L mAb and MECA-79, a mAb that binds to peripheral node addressin, specifically expressed by HEVs, thereby directing the administered nanoparticles to the LN. This strategy allowed marked decreases in anti-CD40L mAb dose and in combination with a few peritransplant doses of rapamycin promoted long-term survival of the heart allografts, where more than 50% of allografts survived more than 60 days in treated recipients (20).

## Clinical implications

Solid organ transplantation is a life-saving procedure performed each year for thousands of patients suffering end-stage organ disease. The immune response to an MHC-mismatched graft is the strongest that can be evoked, necessitating chronic daily administration of immunosuppressive drugs to inhibit donor-reactive responses. The drawbacks of immunosuppressive therapy include increased recipient susceptibility to infection and cancers, and nephrotoxicity specifically from calcineurin inhibitors. The use and harmful effects of these drugs could be avoided by devising efficacious tolerogenic strategies specifically inhibiting graft alloantigen–reactive T cell responses to achieve immunosuppression-free tolerance. Optimized costimulatory blockade agents to achieve immunosuppression-free tolerance to graft-donor alloantigens continues to be an attractive goal and the focus of much investigation in the transplant field. In line with the focus of Zhao, Jung, et al., blockade of CD40-mediated costimulation using CD40L- or CD40-blocking antibodies is likely to present a more effective strategy in achieving long-term allograft tolerance (20, 13). However, initial use of an anti-CD40L mAb in nonhuman primate recipients uncovered an unexpected thromboembolytic effect that negates its clinical use. Newer iterations of anti-CD40L mAb as well as CD40-blocking antibodies are in development and slowly entering the pipeline in transplant therapy (22–24). The results of the studies by Zhao, Jung, and colleagues reveal important mechanistic insights into the required LN site for costimulatory blockade agents. The findings also provide an innovative strategy for administering these antibodies directly to the LN that should circumvent systemic effects and improve graft outcomes in transplant patients (20).

## Figures and Tables

**Figure 1:**
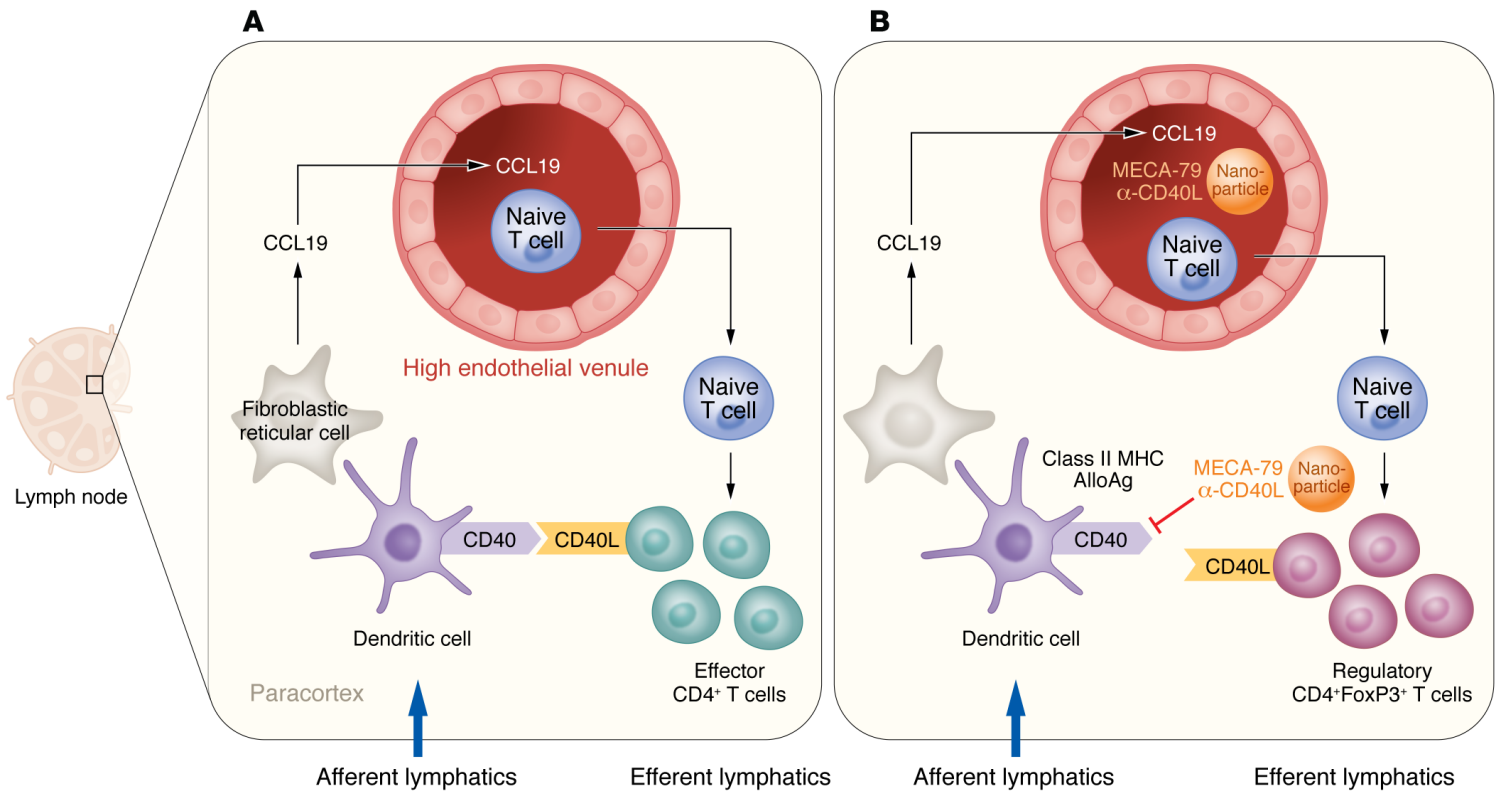
Fibroblastic reticular cells organize alloantigen-reactive effector and regulatory CD4^+^ T cell responses in the lymph node. (**A**) Following allogeneic heart transplantation, fibroblastic reticular cells (FRCs) in the recipient lymph nodes organize interactions between dendritic cells (DCs) entering the lymph nodes through the afferent lymphatics and naive CD4^+^ T cells entering through the high endothelial venules (HEVs). CD4^+^ T cells reactive to graft allogeneic class II MHC molecules and receiving costimulatory signals through engagement of CD40L with DC-expressed CD40 are activated to clonally proliferate and differentiate into effector T cells that will participate in rejection of the allograft. (**B**) Peritransplant treatment with anti-CD40L mAb costimulatory blockade inhibits delivery of the DC CD40-costimulatory signals to the reactive T cells and diverts their differentiation to regulatory cells (Tregs) that inhibit the allograft-reactive immune response and promote long-term allograft acceptance. Zhao and colleagues show that administration of nanoparticles encapsulating the anti-CD40L mAb and conjugated with MECA-79, a mAb directing the beads to and through the HEV, increases the efficacy of the costimulatory blockade treatment in promoting the long-term survival of the allografts.

## Version 1 12/15/2022

Electronic publication
